# Factor Structure of the Ways of Coping Questionnaire in Parkinson's Disease

**DOI:** 10.1155/2018/7128069

**Published:** 2018-12-04

**Authors:** Emily J. Corti, Andrew R. Johnson, Natalie Gasson, Romola S. Bucks, Meghan G. Thomas, Andrea M. Loftus

**Affiliations:** ^1^Curtin Neuroscience Research Laboratory, School of Psychology and Speech Pathology, Curtin University, Perth, WA, Australia; ^2^ParkC Collaborative, Perth, WA, Australia; ^3^School of Psychological Science, University of Western Australia, Perth, WA, Australia; ^4^Experimental and Regenerative Neuroscience, School of Animal Biology, University of Western Australia, Perth, WA, Australia

## Abstract

The Ways of Coping Questionnaire (WCQ) is used extensively in health research, but the measurement properties and suitability of the WCQ for people with Parkinson's disease (PD) have not been psychometrically assessed. If the WCQ does not align with its original 8-factor structure in a PD population, the use of the WCQ subscales may not be appropriate. The present study used confirmatory factor analysis (CFA), exploratory factor analysis (EFA), and multiple-group EFA to determine the ideal factor structure of the WCQ in a PD sample. The original 8 factors of the WCQ were not reproduced. EFA revealed a 6-factor structure, including Distancing, Faith, Avoidance, Seeking Social Support, Planful Problem Solving, and Confrontive coping. As motor symptom severity may impact coping, the stability of the 6-factor structure was examined across motor symptom severity (mild and moderate), remaining consistent. Higher levels of overall motor severity were associated with increased use of faith and avoidance style coping. These findings suggest that the 6-factor structure of the WCQ may be more appropriate for assessing coping styles in PD.

## 1. Introduction

PD is a neurodegenerative disorder characterised by motor (tremor, bradykinesia, and rigidity) and nonmotor symptoms (sleep disturbances, cognitive impairment, depression, anxiety, stress intolerance, and psychiatric dysfunction) that impact significantly upon an individual's functional abilities [1, 2]. People with PD report a range of both illness-specific and general stressors [3], which adversely affect physical and psychological functioning [4, 5]. Stress is thought to play an important role in the development and progression of PD [6, 7]. Recent research indicates that, in a mouse-model, chronic mild stress accelerated motor and nonmotor symptoms [8]. In light of its contribution to the development and progression of PD, coping with stress is a priority in PD.

Coping is the cognitive and behavioural efforts to manage stress [9], typically involving an appraisal of the stressor and the employment of a coping strategy to reduce any negative impact of the stressor [10]. In PD research, stressors have been classified as either emotion- or problem-focussed. Problem-focussed coping strategies (such as Planful Problem Solving) are associated with lower distress and better quality of life in PD [11, 12], whereas emotion-focussed strategies (such as Escape Avoidance or Distraction) are associated with poorer emotional wellbeing and quality of life [11, 13, 14]. It has been suggested that a lack of flexibility in coping processes is associated with poorer mental and physical health outcomes in PD [15], which indicates that some coping choices may be adaptive (i.e., reduce the negative impact of a stressor) for particular stressors/situations yet maladaptive for others. Given its potential to predict emotional wellbeing and quality of life, the accurate assessment of coping strategies in PD is, therefore, important.

The Ways of Coping Questionnaire (WCQ) assesses coping strategies and has been used in a range of healthy populations and chronic conditions (e.g., multiple sclerosis and stroke) [16, 17, 18], including PD [10]. The revised 66-item WCQ asks the participant to think of a recent stressful situation and then requires them to indicate the extent to which they used a particular coping strategy in that situation [10, 19, 20]. Factor analysis of the WCQ-revised revealed eight underlying factors, leading the authors to suggest that the WCQ is comprised of eight “subscales” which map directly onto distinct coping strategies: Planful Problem Solving, Positive Reappraisal, Escape Avoidance, Distancing, Accepting Responsibility, Seeking Social Support, and Self-controlling [19, 20].

Subsequent confirmatory factor analysis (CFA) of the WCQ-revised yielded mixed results, with some findings supporting the eight-factor model [10, 21] and others not [17, 18]. Population differences (clinical versus community samples) and the measurement of coping across different stressors (chronic versus acute; mild versus severe) may have contributed to the mixed results. Lundvist and Ahlström [10] compared the factor structure of the WCQ in a neurological disease sample versus a nonclinical community sample. Using both exploratory and confirmatory factor analyses, only the clinical sample retained the eight-factor structure. This led the authors to suggest that the WCQ may be more suitable for capturing coping strategy use in a clinical sample than a nonclinical sample. This supports Parker et al.'s [17] findings that the 8-factor model could not be reproduced in a university student (nonclinical) sample, using confirmatory or exploratory factor analysis. However, in a clinical population study (multiple sclerosis or spinal cord injury), the eight-factor WCQ was not reproduced using either CFA or EFA [18]. Based on the EFA, the authors instead proposed a three-factor structure, including cognitive reframing, emotional respite, and direct assistance [18]. Furthermore, these three factors did not contain all of the original items from the WCQ, due to poor psychometric fit with the model. Interestingly, this is consistent with Lundvist and Ahlström's [10] findings where 5 of the 66 items were excluded from the final factor structure, despite the eight-factor structure being reproduced. These inconsistencies suggest that the reliability and structure of the WCQ is influenced by sample characteristics. Indeed, a meta-analytic reliability generalisation study reported that sample characteristics significantly impact the reliability of the WCQ subscales, as nonclinical populations may have access to greater and more varied coping resources than clinical populations [4].

In PD, motor severity may impact the likelihood of a person thinking of a PD-specific situation versus a general stressful situation and could impact the coping resources available [11]. In PD, disease severity is positively correlated with perceived stress, suggesting that individuals with increased severity perceive more situations to be stressful in comparison with individuals with lower severity [22]. Frazier [22] suggested that individuals with PD used different coping strategies for different types of stressors (physical, cognitive, and psychosocial). This is consistent with the research that reports individuals with more severe motor symptoms tend to use more emotion-focussed coping strategies [23]. In contrast, use of emotion-focussed coping strategies decreased in individuals with less severe motor symptoms [24]. Taken together, these findings suggest that coping strategies are impacted by disease severity and the type of stressor. Given that physical/motor symptoms are associated with quality of life, activities of daily living, and depression in PD, which in turn impacts on coping resources, it is reasonable to suggest that motor severity may impact the factor structure of the WCQ.

The WCQ-revised is commonly used to assess coping strategies in people with PD [10, 25, 26, 27], but the factor structure of the WCQ has not been thoroughly examined in a PD sample. Whitworth et al. [27] found that 3 of the 8 subscales demonstrated poor internal consistency (Distancing, Confrontive coping, and Accepting Responsibility). These findings suggest that the eight-factor structure of the WCQ may not persist in a PD sample, raising questions about the use of the WCQ subscales as a measure of eight distinct coping strategies in PD. While the WCQ is not the only measure of coping used in PD, no scales have been developed specifically for measuring coping in Parkinson's and few general coping questionnaires have been validated in a PD sample [28]. Therefore, the present study examined the factor structure of the WCQ in a large, community-based PD cohort. Since motor symptom severity may impact upon coping strategies used [22], this study also compared the factor structure of the WCQ-revised in people with mild versus moderate PD motor symptom severity.

## 2. Methods

### 2.1. Participants

Participants were recruited as part of an ongoing study at ParkC in Western Australia. This study was approved by the Curtin University Human Research Ethics Committee. All participants provided written, informed consent. For full testing protocol, see reference [29]. Inclusion in the study required a diagnosis of PD by a geriatrician or neurologist in accordance with the United Kingdom Parkinson's Disease Society Brain Brank Criteria [30]. Two hundred five participants with idiopathic PD were included. As ParkC is a longitudinal study, some (*n* = 146) participants in this study were originally included in Whitworth et al.'s [27] sample. Following the recommendations of Emre et al. [31], no participants demonstrated PD-dementia at study entry. The total MMSE score was calculated using the “World Backwards” subtest [32]: all participants scored ≥25/30, suggesting no global cognitive impairment was present. Sample demographics are reported in Table 1.

## 3. Measures

### 3.1. Coping

The WCQ [19] contains 66 statements about coping with a stressful situation. Participants are asked to think of a stressful situation they experienced in the previous week and are then asked to indicate the extent to which they used a particular coping strategy in that situation (situational specific measurement). Responses are given on a 4-point Likert scale, ranging from 0 to 3 (0 = does not apply and/or not used; 1 = used somewhat; 2 = used quite a bit; and 3 = used a great deal). An example of a Planful Problem Solving coping statement is “made a plan of action and followed it.” Higher scores indicate greater use of that particular coping (compared to not using at all). The factor structure of the WCQ was determined using data from community samples [19, 20, 33]. Eight coping factors were identified using alpha and principal factoring with oblique rotation [34], which map onto 8 distinct coping strategies, including Confrontive coping (6 items), Distancing (6 items), Self-controlling (7 items), Seeking Social Support (6 items), Accepting Responsibility (4 items), Escape Avoidance (8 items), Planful Problem Solving (6 items), and Positive Reappraisal (7 items). The remaining 10 items are distractor items. Internal consistency of the eight subscales ranged from 0.61 to 0.79 (*M* = 0.70 [34]).

### 3.2. Motor Symptom Severity

The Unified Parkinson's Disease Rating Scale-Revised (MDS-UPDRS) is the most commonly used clinical tool for the assessment of PD [35, 36]. Part III of the MDS-UPDRS is a physical examination of motor symptom severity conducted by a trained clinician. 33 different movements are assessed, with severity rated on a scale from 0 (normal) to 4 (severe). The sum of these 33 items was used as the measure of overall motor symptom severity. Scores range from 0 (asymptomatic) to 132 (most severe). For this analysis, participants were classified as having either “mild” or “moderate” motor symptoms (see Table 1 for *N*s), where mild is ≤32 and moderate is ≥33 [37].

### 3.3. Statistical Analysis

Analyses were conducted in four stages using MPlus version 7.4. First, the original 8-factor structure of the WCQ was tested using confirmatory factor analysis (CFA). If the 8-factor structure showed poor fit in the current sample, then exploratory factor analysis (EFA) with geomin rotation (a form of oblique rotation which produces factor loadings and correlations similar to CFA [34, 38]) was used to identify the actual underlying measurement structure. Invariance of that structure between motor severity groups (mild or moderate) was then assessed using multiple-group EFA [39].

To identify possible sources of differences in the measurement of coping, four levels of invariance were tested. Configural invariance tested whether the same factor structure was present for both groups,with no assumption that the components of that structure were the same [39]. Metric invariance assumes that the factor loadings are identical in both groups but allows the item thresholds to differ. Loading invariance was used to test whether the latent constructs had the same effects on the observed items in both groups [40]. Threshold invariance assumes that the item thresholds are the same in both groups, with the factor loadings allowed to differ. Threshold invariance was used to test whether the meaning of each item category was the same for both groups (i.e., whether a response of “used quite a bit” indicated the same amount of use for both groups) [40]. Scalar invariance assumes that both the item thresholds and factor loadings are equal in both groups. Scalar invariance was used to test whether *both* the effects of the latent constructs (factor loadings) and the meaning of each observed item category (item thresholds) were the same for both groups [40].

If the measurement of coping was stable across both mild and moderate motor severity groups, the relationship between motor severity and each of the coping styles was then assessed using exploratory structural equation modelling (ESEM).

All analyses were conducted using Mplus version 7.4. Weighted least squares mean and variance (WLSMV) adjusted estimation with a probit link function was used to account for the ordinal nature of the data. Model fit was assessed using the following: Tucker and Lewis Index (TLI), threshold: >0.90 [40]; the Comparative Fit Index (CFI), threshold: >0.90 [41]; and the Root Mean Square Error of Approximation (RMSEA), threshold: <0.05 [33].

Changes in model fit for invariance testing were assessed using the criteria proposed by Chen [42]. Given the sensitivity of the *χ*^2^ statistic to sample size and non-normality, changes in the CFI and RMSEA statistics have been shown to be more accurate alternatives [41]. A change in CFI (ΔCFI) of ≤0.005 and a change in RMSEA (ΔRMSEA) of ≤0.010 would indicate invariance [42]. For completeness, the *χ*^2^ and WLSMV Δ*χ*^2^ are also reported [43].

## 4. Results

The CFA of the 8-factor structure showed poor fit to the data: CFI = 0.798, TLI = 0.785, and RMSEA = 0.066 (0.061, 0.070). As such, an EFA was then conducted. A 6-factor structure of the WCQ showed the best fit: CFI = 0.96, TLI = 0.94, and RMSEA = 0.03 (0.03, 0.04). Rotated loadings for the 6-factor solution are presented in Table 2. Given their respective items, the factors were labelled as Escape Avoidance problem solving, Confrontive problem solving, Planful Problem Solving, Seeking Social Support, Distancing, and Faith. Five of the identified factors were mapped onto those from the original WCQ 8-factor structure (Escape Avoidance, Confrontive, Planful Problem Solving, Seeking Social Support, and Distancing), while Faith was identified as a new factor in the current study.

The invariance of the 6-factor structure between motor severity groups (mild and moderate) was examined using multiple-group exploratory factor analyses. Each of the models of invariance (configural, loading, threshold, and scalar) showed adequate fit. Moreover, all ΔCFI values were ≤0.005 and all ΔRMSEA values ≤0.010, indicating that measurement invariance was established (Table 3). This suggests that the measurement of coping in PD (conceptualised as the 6-factor model) is not impacted by the overall severity of motor symptoms.

Since the measurement of coping was unaffected by motor severity, the relationship between motor severity and coping could then be assessed without the concern that the results could be due to measurement error. The UPDRS total score (indicative of motor severity) was entered as a predictor of the 6 coping factors using ESEM. Significant, positive relationships were observed for avoidance coping (standardised *B* = 0.232, *p*=0.001) and faith coping (standardised *B* = 0.166, *p*=0.039). This indicates that individuals with more severe motor symptoms reported using more avoidance and faith-based coping, but there was no effect of PD motor symptom severity on the other 4 coping strategies.

## 5. Discussion

The present study used CFA, EFA, and multiple-group EFA to examine the factor structure of the WCQ in a PD sample. The original, eight-factor structure was not reproduced in this cohort. The present analysis identified six factors; Escape Avoidance, Faith, Confrontive, Seeking Social Support, Planful Problem Solving, and Distancing. While five of the factors map to some of the original 8 factors, only 2 did so closely and one new factor was identified; Faith. Furthermore, the 6-factor model identified in this study was consistent across mild and moderate motor symptom severity. In addition, results also indicated that overall motor symptom severity predicted the use of Escape Avoidance and Faith-based coping, such that greater symptom severity was associated with greater avoidance and faith-based coping.

In the six-factor model of the WCQ found in this cohort of people with PD, only 2 of the original 8 factors were substantively reproduced [20]. These are Seeking Social Support and Planful Problem Solving. All original items for Seeking Social Support, excepting one, loaded onto the factor. Item 22 (“I got professional help”) did not load onto the Seeking Social Support factor, or indeed any factor. Planful Problem Solving was reproduced in the same manner as the original eight-factor model, with some additional items mapping onto this factor (“stop it interfering with other things,” “talk to find out more,” and “talked to someone who helped”).

The three remaining factors (Confrontive, Distancing, and Escape Avoidance) share similarities with the original eight-factor model but were not entirely reproduced in the present study. Two original items loaded onto the Confrontive factor, alongside an additional item “took it out on others.” Whilst item 22 did not load onto any factor in the original structure, this item is consistent with the definition of Confrontive coping that encompasses aggression and hostility. In the present study, the Distancing and Escape Avoidance scales loaded interchangeably, such that items from the original WCQ Distancing factor loaded onto the Escape Avoidance factor in the current study, and vice versa. The Distancing factor related to detaching oneself from the stressful situation, while the Avoidance factor related to avoiding the stressful situation. This suggests that the current PD sample may see Distancing and Escape Avoidance as related coping strategies. These differences suggest that the original eight-factor structure of the WCQ may not adequately capture coping strategies in a PD sample. The lack of clear presentation of the two remaining factors (Accepting Responsibility and Self-Control) suggests that people with PD are unlikely to use these coping strategies.

The items that formed the new factor identified (Faith) are all from the original, Positive Reappraisal factor. In the WCQ, Positive Reappraisal describes using personal growth to create positive meaning [20]. It also contains a religious dimension (e.g., “I prayed” and “found new faith”). Only the religious items loaded on this factor, and as such, they were identified as a new factor in the present analysis. Research has found that increased age of onset and duration of disease seemed to be related to an increased use of religious coping in Parkinson's [44, 45]. It would be interesting to investigate in future research whether self-reported religiosity is related to the use of these coping strategies.

The two original factors not reproduced in the present study are Accepting Responsibility and Self-Control. The lack of clear presentation of these factors suggests that people with PD are unlikely to use these coping strategies. It is reasonable to suggest that these factors are not appropriate in a PD population, due to the idiopathic nature of the disease, the fact that there is no cure for PD, and the contextual nature of the WCQ (i.e., the participant is required to think of a specific stressful event). It is possible that the sample in this study, when asked to think of a stressful event, thought of a PD-specific stressor, such as freezing in public. Such a stressor is often beyond the control of the individual, and coping strategies such as Self-Control and Accepting Responsibility are, therefore, redundant. This is consistent with studies in other clinical populations, which suggest that some coping strategies are not relevant in many clinical conditions and are therefore not captured using the 8-factor WCQ [4, 18]. In addition, people with moderate motor symptoms used more Faith and Escape Avoidance coping compared to those with mild motor symptoms. The greater use of Escape Avoidance and Faith-based coping in those with more severe motor symptoms provides further evidence that participants may be responding to the questionnaire while thinking of a stressful situation out of their control such as a situation relating directly to their reduced motor function.

Understanding the way an individual with PD copes with stressful situations is important for identifying adaptive coping strategies in PD. Greater motor severity is associated with an increased use of potentially maladaptive coping strategies (Faith and Avoidance), which have historically been linked to poor health-related quality of life [15, 46]. In comparison, active coping strategies are associated with greater quality of life and less perceived stressful situations [12, 15]. Identifying those situations where active coping strategies serve to reduce perceived stress may go some way to improving overall quality of life in PD.

The present study revealed that the original eight-factor structure of the WCQ did not accurately capture coping strategies used by people with PD and highlights that coping in situations where people have no control over a situation (such as an incurable neurological condition) may potentially be different from coping in everyday situations. As suggested by Whitworth et al. [27], the context in which individuals apply coping strategies must be considered in the actual measurement of coping. In particular, it is important to delineate coping strategies used in PD-specific and non-PD-specific stressful situations. The selection of context-specific coping questionnaires should be considered in both research and clinical assessment. By directing people with PD to think of either a PD-specific or non-PD-specific situation, we will gain a better understanding of the use of coping strategies.

## Figures and Tables

**Table 1:** Sample demographics (means and standard deviations).

	Full sample (*N* = 205)	Mild severity (*N* = 114)	Moderate severity (*N* = 91)
Sex			
** **Male	140	78	62
** **Female	65	36	29
Age (years)	65.72 (9.73)	63.30 (9.31)	68.85 (9.27)
UPDRS part 3 total (max. 132)	31.46 (14.16)	21.53 (7.11)	43.91 (10.45)
Disease duration (months)	70.28 (60.77)	61.52 (51.38)	81.76 (70.52)
LED	616.60 (458.66)	619.05 (426.01)	611.23 (496.35)

*Note.* LED = levodopa equivalent dose [33].

**Table 2:** Rotated loadings for 6-factor exploratory factor analysis.

	Escape Avoidance	Confrontive	Planful Problem Solving	Seeking Social Support	Distancing	Faith
Criticised/lectured myself	**0.630** ^*∗*^					
Tried not to burn bridges	0.336^*∗*^					
Hoped for miracle^+^	0.568^*∗*^					
Went along with fate/luck	**0.650** ^*∗*^					
Slept more^+^	**0.597** ^*∗*^					
Tried to forget	0.534^*∗*^					
Avoided others^+^	**0.399** ^*∗*^					
Refused to believe^+^	**0.535** ^*∗*^					
Wished situation would go away^+^	**0.564** ^*∗*^					
Fantasies^+^	0.457^*∗*^					
Tried to change person's mind^+^		**0.707** ^*∗*^				
Expressed anger^+^		**0.843** ^*∗*^				
Took it out on others		**0.684** ^*∗*^				
Concentrate on next step^+^			0.504^*∗*^			−0.353^*∗*^
Inspired to be creative			**0.508** ^*∗*^			
Made plan of action^+^			**0.682** ^*∗*^			
Changed something^+^			**0.567** ^*∗*^			
Drew on past experience^+^			0.572^*∗*^		0.304	
Double efforts to work^+^			**0.774** ^*∗*^			
Couple of solutions^+^			**0.778** ^*∗*^			
Stop it interfering with other things			0.336^*∗*^			
Talk to find out more^+^			0.353	0.524^*∗*^		
Talked to someone who helped^+^			0.396	0.530^*∗*^		
Look for silver lining				0.316		
Accepted sympathy/understanding^+^				**0.724** ^*∗*^		
Asked advice^+^				**0.754** ^*∗*^		
Talked to someone^+^				**0.716** ^*∗*^		
Brought the problem on myself					0.392^*∗*^	
Kept others from knowing					**0.478** ^*∗*^	
Made light of situation^+^					**0.628** ^*∗*^	
Didn't let it get to me^+^					0.618^*∗*^	−0.317^*∗*^
Found new faith				0.327		**0.753** ^*∗*^
Changed myself						0.378^*∗*^
Prayed				0.328		0.632^*∗*^

*Note.* Loadings ≤0.3 suppressed; bold indicates a loading twice that or greater than the loading on any other factor. ^+^Item loads onto the same factor in the original 8-factor structure WCQ.^*∗*^*P* < .05.

**Table 3:** Invariance testing model fit statistics.

Model	*χ* ^2^ (df)	Δ*χ*^2^ (*df*)	CFI	ΔCFI	RMSEA (90% CI)	ΔRMSEA	TLI
Configural	2031.08 (1880)^*∗*^		0.964		0.028 (0.016, 0.037)		0.953
Loading	2315.67 (2144)^*∗*^	323.79 (264)^*∗*^	0.959	0.005	0.028 (0.017, 0.036)	−0.001	0.954
Threshold	2136.26 (1980)^*∗*^	116.15 (100)	0.963	0.001	0.028 (0.016, 0.037)	−0.001	0.954
Scalar	2435.08 (2244)^*∗*^	336.289 (264)^*∗*^	0.955	0.004	0.029 (0.018, 0.037)	0.003	0.951

*Note.* ^*∗*^ *p* ≤ 0.05, ΔCFI = change in CFI (ΔCFI ≤0.005 indicates invariance), ΔRMSEA = change in RMSEA (ΔRMSEA ≤0.010 indicates invariance).

## Data Availability

The data used to support the findings of this study may be released upon application to the ParkC Executive Committee, by contacting Associate Professor Andrea Loftus at School of Psychology, Curtin University, GPO Box U1987, Perth, WA 6845; andrea.loftus@curtin.edu.au.
